# Low-Altitude Boundary of *Abies faxoniana* Is More Susceptible to Long-Term Open-Top Chamber Warming in the Eastern Tibetan Plateau

**DOI:** 10.3389/fpls.2021.766368

**Published:** 2021-12-03

**Authors:** Haifeng Song, Qingquan Han, Sheng Zhang

**Affiliations:** Key Laboratory of Bio-Resource and Eco-Environment of Ministry of Education, College of Life Sciences, Sichuan University, Chengdu, China

**Keywords:** warming, OTC, altitude, metabolism, adaption, sub-alpine

## Abstract

With global climate change, for evaluating warming effect on subalpine forest distribution, the substantial effects of long-term warming on tree growth and soil nutrients need to be explored. In this study, we focused on different responses in the boundaries of trees and soils to warming. Using the open-top chamber (OTC), a 10-year artificial warming experiment was conducted to evaluate the impacts of warming on *Abies faxoniana* at three different altitudes. We determined metabolites and nutrient concentrations in needles of *A. faxoniana* and characterized the soil chemistries. Many kinds of sugars, amino acids, and organic acids showed higher contents at high altitude (3,500 m) compared with low altitude (2,600 m), which could have been due to the temperature differences. Warming significantly decreased needle sugar and amino acid concentrations at high altitude but increased them at low altitude. These results indicated contrasting physiological and metabolic responses of *A. faxoniana* to long-term warming at different altitudes. Furthermore, we found that OTC warming significantly increased the concentrations of soil extractable sodium, aluminum (Al), and manganese (Mn), while decreased potassium (K) and phosphorus (P) concentrations and pH values at low altitude rather than at middle (3,000 m) or high altitude. The soil carbon and nitrogen contents were increased only at the middle altitude. In *A. faxoniana* at low altitudes, more mineral nutrients iron, K, and P were demand, and a mass of Al, Mn, and zinc was accumulated under warming. Soil P limitation and heavy metals accumulation are disadvantageous for trees at low altitudes with warming. Therefore, compared with high altitudes, *A. faxoniana* growing at low boundary in alpine regions is expected to be more susceptible to warming.

## Introduction

Global warming has attracted much attention around the world. The Tibetan Plateau is experiencing a higher warming rate than elsewhere in the Northern Hemisphere (Duan and Xiao, 2015; Guo et al., 2018), where a 0.32°C increase per decade has occurred over the past 50 years (Luo et al., 2013; Wei and Fang, 2013). Mountain forests are of particular importance in maintaining regional ecosystem stability and succession in China. Temperature acts as a key factor in regulating plant physiology (Rustad et al., 2001; Ziello et al., 2009). Altitudinal gradient is closely related to plant physiology because of a vertical temperature range (Körner, 2007). The rate of warming in mountain forests is amplified with elevation (Pepin et al., 2015). Alpine forests might be especially susceptible to warming, and the sensitivity would vary with elevation (Wang et al., 2017; Panthi et al., 2020). There are many studies on the sink-source balance by exploring alterations in plant growth, photosynthesis, and respiration under experimental warming (Yin et al., 2008; Volder et al., 2013; Harvey et al., 2020; Carter et al., 2021). However, few studies have focused on leaf phenotypic plasticity and nutrient traits combined with soil properties, in long-term field warming experiments.

Many plant physiological processes and traits can be affected by temperature alteration, such as biosynthesis of carbohydrates and leaf nutrient accumulation (Aspinwall et al., 2016). Temperature alteration can damage in the plant cells by disturbing the balance in cellular respiration. Soluble carbohydrates and amino acids play important roles in protecting plant photosynthetic apparatus to maintain photosynthetic capacity, cellular osmotic homeostasis, and membrane stability. A study on *Solanum tuberosum* revealed that increased temperature could increase the concentration of many primary metabolites including amino acids, fatty acids, fatty alcohols, and organic acids (Hancock et al., 2014). In addition, warming also induced alterations in the nutrient availability in soil and plant nutrient absorption capacity in soils and plants (D’Orangeville et al., 2014; Wu et al., 2020). Previous studies have reported that the effects of warming on soil properties are closely related to tree physiology in subalpine regions (Yin et al., 2013; Zhao et al., 2014). Short-term field warming could change soil microbial carbon (C), nitrogen (N), and the microbial community structure, and there were different physiological responses on the growth of *Abies faxoniana* seedlings (Wu et al., 2011; Xu et al., 2013, 2015). However, the effect of soil change on tree physiology responding to long-term warming is still unknown.

Previous studies have found that *A. faxoniana* seedlings from higher altitude have higher leaf N status, net photosynthetic rate, and greater non-structural carbohydrate concentrations than those from lower altitude (Wu et al., 2011; Ran et al., 2013). Phenolic compounds and mineral contents are also varied across altitudes under warming (Wu et al., 2011; Ran et al., 2013; Stromme et al., 2018; Mason et al., 2019). Trees at high elevation are expected to have local adaptation to high frequency and intensity of cold events. The concentrations of sugars, organic acids, and amines would be increased more strongly in high than low-altitude populations (Schrieber et al., 2020). However, tree species in the low treeline ecotones have been found to be more sensitive to climatic factors (Takahashi et al., 2003; Kueppers et al., 2017). Evidence have shown that tree growth declines and mortality increases at lower altitudes as temperature increases (Huang et al., 2017; Wang et al., 2017), whereas at higher altitudes, tree growth and tree density increase synchronously (Liang et al., 2011; Dang et al., 2012; Ponocna et al., 2016; Conlisk et al., 2017).

Temperature is a dominant constraint on tree growth in the high elevations on the eastern Tibetan Plateau (Wang et al., 2017). The growth of firs in subalpine regions in the eastern Tibetan Plateau is sensitive to changed temperature regime rather than the variation of precipitation pattern (Wang et al., 2017). The plant metabolome is the chemical phenotype of individual plants or populations under specific environments (Rivas-Ubach et al., 2012). Additionally, for long-living trees, genetic adaptation is slow (Savolainen et al., 2004). Therefore, they are likely to cope with environmental changes more rapidly through plastic responses than through microevolution (Chevin et al., 2013; Franks et al., 2014). Trees are likely to have unique metabolic profiles due to long-term warming at different altitudes, particularly in perennial forests (Dhuli et al., 2014). However, how much phenotypic plasticity can help them cope with climate change remains largely unknown (Anderson et al., 2012; Duputie et al., 2015). It is important to understand how tree physiology and growth will respond to global changes on a wide scale.

In the present study, subalpine forest systems present unique opportunities to study the warming effects across different altitudes because they are characterized by different temperatures. To explore tree physiological responses to warming, the metabolome and ionome analyses were performed to investigate the responses of trees to a 10 year long-term field warming. We have performed artificial warming experiments using open-top chambers (OTCs) with *A. faxoniana* in this region since 2007 (Wu et al., 2011). We will address the following hypotheses: (1) long-term warming can induce the different alterations in mineral nutrients and metabolites of *A. faxoniana* at high and low altitudes; and (2) the low-altitude boundary of *A. faxoniana* is more susceptible to warming than the high-altitude.

## Materials and Methods

### Experimental Site Description

The experiments were conducted at the Wanglang National Nature Reserve (32°49′–33°02′N, 103°55′–104°10′E), which is located in the eastern Tibetan Plateau, China. This region is in the monsoon climate zone, and there are distinct dry (November to April) and wet (May to October) seasons. The maximum and minimum temperatures in summer and winter are 26.2 and −17.8°C, and the mean annual temperature is from 5.07 to 1.57°C. The annual mean precipitation ranges from 801 to 825 mm, with most rainfalls happening from May to August (Ran et al., 2013). According to the Chinese soil classification (Chinese Soil Taxonomy Research Group, 1995), the soil type is mountain dark brown forest soil (Tang et al., 2019). *A. faxoniana* is a dominant tree species and the natural distribution ranges from 2,600 to 3,500 m on shady slopes in this region (Ran et al., 2013). The forest canopy density was 0.80–0.85 with a stand density of 810–900 ha^–1^ of pure fir trees.

### Experimental Design and Sampling

The *in situ* experiments were designed in early September 2007. To provide an effective and simple method of warming simulation in the field for maintaining precipitation, light, and gas exchange closest to natural levels, OTCs, a passive warming method, were used for above-ground systems warming in this study. OTC warming is a low-maintenance and cost-effective method for field experiments across environmental gradients or in remote areas, especially in alpine area (Marion et al., 1997; Walker et al., 2006). Sites used for OTC are with similar canopy (density of 0.85) and slope angles (35–40°). OTCs are located in open areas where rain and snow could fall into. The OTCs used in this study are 2 m high, with a ground area of 2.25 m^2^, tapering to 1.5 m^2^ at the open top. The materials are made of solar-transmitting material (8-mm thick acrylic fiber). The walls of the OTCs have a 35–40° inclination relative to the ground. It ensures that the raining water can fall into the OTC from the open tops and interstitial flow from the inclination. Just like most published papers (Xu et al., 2013; Descombes et al., 2020), each site at any elevation included six OTCs and six control plots, respectively. Control plots were established near the OTC plot at each site. Ten-year-old *A. faxoniana* saplings (35 ± 5 cm height) were transplanted into 18 OTCs and 18 ambient plots (controls) at altitudes of 2,600, 3,000, and 3,500 m, respectively (Supplementary Figure 1A). In order to minimize the difference in field, saplings were collected from the forest gaps of the corresponding elevations to make them have similar genetic background. Five saplings were included in each OTC or control plot (1.5 m × 1.5 m). Four saplings were planted with a distance of 0.5 m and one sapling was planted in the middle (Supplementary Figure 1B). Finally, a total of 60 saplings (30 warming and 30 control individuals) were included at each altitude. The distance between each subplot is more than 10 m. Six TP-2200 humidity and temperature loggers (Fotel, Shanghai, China) were installed into the experimental plots to measure the temperature and moisture (Xu et al., 2013, 2015). Twice completely interannual temperature and moisture data (from January to December) were collected in 2015 and 2016, respectively. Because the mean annual temperature did not deviate too much from 2015 to 2016, we used the mean temperature of the 2 years as a proxy for the temperatures during the experiment period (Table 1 and Supplementary Figure 2).

**TABLE 1:** The details of experimental plots and treatments.

**Sites**	**Longitude (°E)**	**Latitude (°N)**	**Annual air temperature (°C)**			**Annual air humidity (%)**		
			**CK**	**OTC**	**ΔT**	**CK**	**OTC**	**ΔH**
3,500 m (H1)	104.03	32.98	1.52 ± 0.51	2.09 ± 0.32	0.57 ± 0.13	88.25 ± 3.2	86.50 ± 5.35	−1.75 ± 0.12
3,000 m (H2)	104.03	33.00	2.87 ± 0.46	3.56 ± 0.40	0.69 ± 0.06	90.05 ± 4.71	87.34 ± 6.72	−2.71 ± 0.17
2,600 m (H3)	104.09	32.97	5.07 ± 0.63	6.11 ± 0.31	1.04 ± 0.25	85.12 ± 6.03	82.01 ± 6.20	−3.11 ± 0.09

*CK, ambient conditions; OTC, open top chambers; ΔT, changed annual temperature; ΔH, changed annual humidity. These data was the mean on January to December 2015 and 2016.*

After 10 years, on August 26, 2017, only 1-year-old needles from the middle-planted sampling of each plot were harvested at 10:00–13:00, randomly divided into five parts. In order to wash out the inorganic matters from the air and fallen organic matters in the needles, they were washed by deionized water. Then, each part of the needles was wrapped using silver paper and stored into liquid nitrogen immediately for further analyzing. For the soil collection, 15 soil cores (5 cm in diameter) of 5–10 cm depth were taken from each temperature regime at each altitude. The three soil cores from each plot were combined to form one composite sample. Because one control and two OTCs chambers were destroyed by fallen trees at the altitude of 2,600 m, four biological replicates were used for 2,600 m and five biological replicates for 3,000 and 3,500 m, respectively.

### Determination of Height and Needle δ^13^C Values

Plant height and diameter were measured on August 25, 2017. The abundance of stable carbon isotopes in the needle samples was determined with an isotope mass spectrometer (Finnegan MAT Delta V Advantage, Bremen, Germany). Needle samples were oven-dried at 70°C for 48 h. Dried samples (0.20 g) were finely ground, and the relative abundances of ^13^C and ^12^C were tested according to Chen et al. (2014). The carbon isotope composition was expressed as δ^13^C value. The overall precision of the δ values was 0.1‰, as determined by the repetitive samples. The δ^13^C of needle samples was described relative to the standard Pee Dee Belemnite as follow method: δ^13^C = (*R*_sample_/*R*_standard_ − 1) × 1,000. *R*_sample_ means the ^13^C/^12^C ratio of samples, and *R*_standard_ that of standard (Farquhar et al., 1989).

### Measurement of Mineral Nutrients in Needles and Soils

Needles and soils were dried at 80°C, and 0.20 g of samples was burned off organic matter using a muffle furnace (450°C). Tissue nutrient concentrations were determined by ash digestion (500°C for 6 h) dissolved in 10 ml of nitric acid and peroxide. Dried soil samples were also digested by nitric acid. The digested plant and soil materials were used to determine mineral nutrient concentrations of calcium (Ca), sodium (Na), aluminum (Al), manganese (Mn), iron (Fe), zinc (Zn), titanium (Ti), chrome (Cr), K, Mg, and P by an Agilent 710 ICP-OES spectrometer (Agilent, Palo Alto, CA, United States) according to instructions (Se et al., 2019; Tang et al., 2019). The total C and N concentrations were analyzed with an elementar vario MACRO cube (Elementar, Germany) (Zuo et al., 2016).

### Metabolite Extraction From Needles of *Abies faxoniana*

The extraction of needle metabolites was performed according to Zhang et al. (2019). Exactly 0.15 g of frozen 1-year-old needles of *A. faxoniana* was ground in a mortar under liquid nitrogen. The metabolites were extracted using 100% precooled methanol (1,500 μl) and vortexed for 30 s. Then, 60 μl ribitol (0.2 mg ml^–1^) was added as the internal quantitative standard. Subsequently, they were ultrasonically processed for 15 min, and 750 μl of chloroform (−20°C) and 1,500 μl of dH_2_O (4°C) were added. After centrifugation at 4,000 × *g* for 15 min, the supernatants were transferred into new tubes. They were dried in a Termovap sample concentrator (DC150-1A, Yooning Instrument, Hangzhou, China). Then, 60 μl of methoxyamine hydrochloride in pyridine (15 mg ml^–1^) was added, and the mixture was incubated at 38°C for 16 h. Finally, 60 μl of Bis(trimethylsilyl)trifluoroacetamide (BSTFA), including 1% chlorotrimethylsilane, was added to the mixture and incubated for 60 min at room temperature.

### GC–MS and Metabolic Profile Analysis

Metabolites were determined using non-targeted metabolomics with an Agilent 7890A GC/5975C MS system (Agilent, California, Palo Alto, United States). One microliter of extract was injected into a 5% phenyl methyl silox of an HP-5 MS capillary column with a 20:1 split ratio. The columns were 0.25 μm df and 30 m × 0.25 mm ID with 10 m Integra-Guard (Agilent J and W scientific, Folsom, CA, United States). The MS source temperature was 250°C, the MS quad temperature was 150°C and the injection temperature was 280°C. After an initial isothermal heating at 80°C for 5 min, there was a 20°C min^–1^ ramp-up to 300°C followed by heating for 6 min. Helium (1.0 ml min^–1^) was used as the carrier gas. The total running time was 22 min. Using a full-scanning method, mass spectrometry was conducted at a range from 35 to 780 (m/z). The corresponding retention times, mass charge ratio and resolution of peaks were resolved by AMDIS software and compared with the NIST^1^ commercial database according to Behrends et al. (2011). In this study, only one salinization substance (TMS) was used for analysis. The metabolic pathway was constructed by combining the KEGG metabolic database^2^.

We used XCMS software^3^ for raw signal extraction, data filtering and peak identification. The setting parameters were as follows: XCMS Set (snthresh = 3, fwhm = 3, mzdiff = 0.5, steps = 2, step = 0.1, max = 300) and retcor [plottype = c (deviation), method = obiwarp, minfrac = 0.3, and bandwidth (bw) = 2]. The final data were imported into Excel tables for multiple linear regression analyses. A quantitative compound (QC) was used to remove misassignment and background noise and to ensure the accuracy of the identified true chemical entities. Before statistical analysis, the total mass of the signal integration area was used to normalize each sample. Then, the normalized data were imported into SIMCA-P 11.0 software (Umetrics AB, Umea, Sweden). Finally, two-way ANOVA was used to identify the effects of altitude, warming, and their interaction. Only metabolites with *p* < 0.05 and the fold change >1.2 were considered as differentially expressed metabolites (DEMs), according to Yokoi et al. (2015).

### Statistical Data Analyses

To detect the difference among treatments, ANOVA with Tukey’s test at the *p* < 0.05 level was used for identifying significant differences with the SPSS 16.0 software (SPSS, Inc., Chicago, IL, United States). The metabolites were standardized using SIMCA-P 11.0^4^. To better understand the differences between the ambient and OTC warming treatments, *t*-tests (*p* < 0.05) were performed within altitudes. In order to reduce differences between groups and make a better separation between groups, partial least squares discrimination analysis (PLS-DA) was performed to display the variation patterns in all the metabolites data instead of principal component analysis (PCA). Orthogonal partial least squares discrimination analysis (OPLS-DA) was conducted to demonstrate the alteration caused by OTC in each attitude. Matrix scatter plot was performed by the package GGally in R. The Pearson’s product–moment correlation coefficients between metabolites and the element contents were calculated and visualized in a heatmap. MetaboAnalyst 4.0 software^5^ was used to construct a heat map diagram of the metabolite categories (Chong et al., 2018).

## Results

### Soil Mineral Nutrient Changes in Response to Open-Top Chamber Warming at Three Altitudes

Under ambient conditions, the concentrations of Ca, Na, Al, Mg, and K decreased with a descending elevation gradient (Table 2). OTC warming significantly increased the concentrations of Na, Al, Mn, Zn, and the ratios of C:P and N:P, but decreased the concentrations of Mg, K, and P in soils at 2,600 m. However, it significantly decreased Ca, Na, Zn, Al, and Mg concentrations at 3,500 m. Soil C and N concentrations were significantly increased by OTC warming only at 3,000 m.

**TABLE 2:** Soil element contents (g Kg^–1^) in the ambient and open top chambers (OTC).

**Treatments**	**H1**		**H2**		**H3**		***p*_*t*_**	***p*_*a*_**	***p*_*t*__×_ *_*a*_***
	**CK**	**OTC**	**CK**	**OTC**	**CK**	**OTC**			
pH	6.17 ± 0.20*c*	5.67 ± 0.13*abc*	5.64 ± 0.11*abc*	5.35 ± 0.10*ab*	5.89 ± 0.11*bc*	5.23 ± 0.14*a*	0.011	0.002	0.883
Ca (g Kg^–1^)	93.08 ± 1.62*c*	51.75 ± 12.82*ab*	48.56 ± 4.69*ab*	92.87 ± 20.46*c*	34.44 ± 6.11*a*	36.78 ± 4.85*a*	0.001	0.334	0.037
Na (g Kg^–1^)	6.69 ± 0.26*c*	4.90 ± 0.32*b*	6.08 ± 0.22*bc*	2.94 ± 0.26*a*	2.90 ± 0.31*a*	6.02 ± 0.49*bc*	0.568	0.000	0.041
Al (g Kg^–1^)	20.05 ± 0.70*b*	12.91 ± 1.22*a*	16.12 ± 1.56*ab*	14.47 ± 0.61*a*	16.21 ± 0.58*ab*	35.08 ± 1.35*c*	0.000	0.000	0.000
Mg (g Kg^–1^)	6.60 ± 0.38*d*	5.03 ± 0.41*c*	5.43 ± 0.35*cd*	2.96 ± 0.23*b*	5.30 ± 0.34*cd*	1.24 ± 0.12*a*	0.006	0.000	0.015
K (g Kg^–1^)	12.25 ± 0.89*b*	9.64 ± 0.87*ab*	11.66 ± 0.69*b*	6.17 ± 0.31*a*	10.54 ± 1.20*b*	6.30 ± 0.30*a*	0.818	0.000	0.096
Fe (g Kg^–1^)	18.77 ± 1.08*ab*	16.97 ± 1.59*ab*	19.02 ± 2.01*b*	11.75 ± 0.86*a*	15.32 ± 0.76*ab*	16.18 ± 2.05*ab*	0.422	0.021	0.140
Mn (g Kg^–1^)	0.37 ± 0.04*a*	0.44 ± 0.08*a*	0.37 ± 0.09*a*	0.38 ± 0.12*a*	0.40 ± 0.02*a*	0.91 ± 0.15*b*	0.026	0.012	0.006
Zn (g Kg^–1^)	0.10 ± 0.007*c*	0.07 ± 0.010*b*	0.07 ± 0.007*b*	0.08 ± 0.015*b*	0.05 ± 0.006*a*	0.08 ± 0.014*b*	0.061	0.051	0.500
Ti (g Kg^–1^)	3.16 ± 0.17*ab*	2.75 ± 0.20*ab*	3.24 ± 0.19*ab*	2.17 ± 0.72*a*	4.13 ± 0.07*b*	2.64 ± 0.31*ab*	0.042	0.768	0.013
Cr (g Kg^–1^)	0.05 ± 0.002*ab*	0.04 ± 0.003*ab*	0.05 ± 0.004*ab*	0.03 ± 0.003*a*	0.05 ± 0.003*b*	0.04 ± 0.005*ab*	0.093	0.458	0.012
C (g Kg^–1^)	97.75 ± 12.28*a*	104.94 ± 22.48*a*	116.09 ± 3.40*a*	245.76 ± 22.12*b*	135.62 ± 11.14*a*	126.36 ± 23.45*a*	0.016	0.003	0.015
N (g Kg^–1^)	6.47 ± 0.74*a*	7.78 ± 1.10*a*	7.90 ± 0.15*a*	14.61 ± 0.96*c*	8.90 ± 0.52*ab*	9.14 ± 0.94*b*	0.011	0.002	0.015
P (g Kg^–1^)	1.09 ± 0.15*c*	0.83 ± 0.13*abc*	0.71 ± 0.07*abc*	0.59 ± 0.03*ab*	1.01 ± 0.07*bc*	0.46 ± 0.07*a*	0.171	0.001	0.962
C:N	15.11 ± 2.58*a*	13.49 ± 1.07*a*	14.70 ± 0.32*a*	16.77 ± 0.55*a*	15.21 ± 0.68*a*	13.61 ± 1.12*a*	0.402	0.410	0.572
C:P	89.68 ± 27.32*a*	126.43 ± 25.01*ab*	167.52 ± 17.32*b*	412.00 ± 15.91*d*	134.27 ± 29.28*ab*	274.70 ± 77.18*c*	0.459	0.000	0.343
N:P	5.94 ± 0.77*a*	9.37 ± 1.06*ab*	11.37 ± 0.99*b*	24.56 ± 0.31*d*	8.81 ± 1.38*ab*	19.87 ± 0.85*c*	0.768	0.000	0.493

*Values denoted the different letters indicate significance at *p* < 0.05 according to Tukey test. *P*_*a*_, altitude effect; *P*_*o*_, OTC effect; *P*_*a* ×_ *_*o*_*, altitude and OTC interaction effect; CK, ambient conditions. H1, 3,500 m; H2, 3,000 m; H3, 2,600 m.*

### Changes in Physiological Traits of *Abies faxoniana* in Response to Open-Top Chamber Warming at Three Altitudes

**FIGURE 1:**
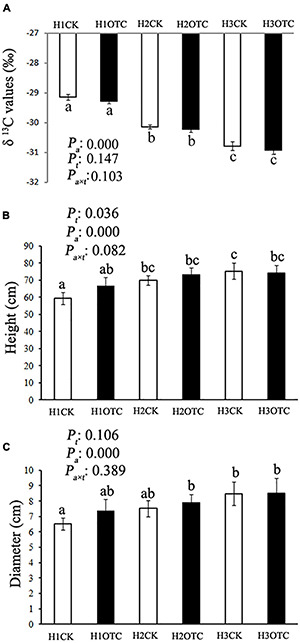
The needle δ^13^C values **(A)** and the growth of height **(B)** and diameter **(C)** of *A. faxoniana* at 3,500, 3,000, and 2,600 m after 10-year warming. Values denoted the different letters indicates a significance at *p* < 0.05 according to Tukey test. *P*_*a*_, altitude effect; *P*_*t*_, open-top chamber (OTC) effect; *P*_*a* ×_ *_*t*_*, altitude and OTC interaction effect; H1CK, 3,500 m control; H1OTC, 3,500 m OTC warming; H2CK, 3,000 m control; H2OTC, 3,000 m OTC warming; H3CK, 2,600 m control; H3OTC, 2,600 m OTC warming.

The effect of altitude on δ^13^C values in needles was significant, showing lower values along a descending elevation gradient (Figure 1A). OTC warming did not significantly change needle δ^13^C values at any altitude. The growth of height and diameter increased with decreasing altitude (Figures 1B,C), and there was a significant difference between altitudes of 2,600 and 3,500 m (*P*_*a*_ < 0.05). However, OTC warming did not cause significant change in the height and diameter of *A. faxoniana* at any altitude.

**FIGURE 2:**
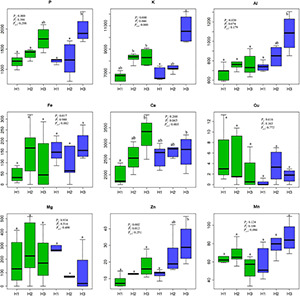
Mineral nutrient contents (μg g^–1^ Dw) in needles of *A. faxoniana* at 3,500, 3,000, and 2,600 m under controls and OTCs. Values denoted the different letters indicates a significance at *p* < 0.05 according to Tukey test. *P*_*a*_, altitude effect; *P*_*t*_, OTC effect; *P*_*a* ×_ *_*t*_*, altitude and OTC interaction effect; H1, the altitude of 3,500 m; H2, the altitude of 3,000 m; H3, the altitude of 2,600 m; the green bars indicate the control conditions; the blue bars indicate the OTC warming conditions.

**FIGURE 3:**
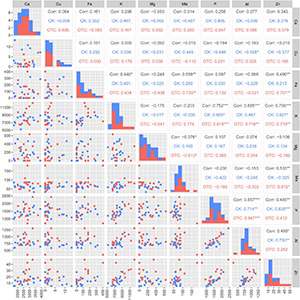
Scatter matrix plot: relationship between needle elements in CK groups at the three attitudes, OTC groups at the three attitudes, and all groups include CK groups and OTC groups at the three attitudes. *0.01 < *P* ≤ 0.05; **0.001 < *P* ≤ 0.01; ***P < 0.001.

Under ambient conditions, the needle Ca and K concentrations were significantly higher at 2,600 m than at 3,500 m (Figure 2). The concentrations of P, Al, and Zn showed increased tendencies with the decline of elevations. OTC warming significantly increased the concentrations of K, Al, and Zn in needles at 2,600 m. The overall effect on element concentration is not significant, but *p* < 0.05 (*P*_*a*_) was observed in some elements like P, K, Al, Fe, and Zn. Therefore, we further explored elements correlation, and heatmap cluster analysis for needle ion contents. As shown in Figure 3, the correlations of P with K, Mn, Fe, and Al were changed by warming. Figure 3 and Supplementary Figure 3A showed a clearly changed tendency of the needle nutrients. Among these, P, Al, K, and Zn clustered into the same category and increased with the increased temperature.

### Metabolic Changes in Responses to Open-Top Chamber Warming at Three Altitudes

**FIGURE 4:**
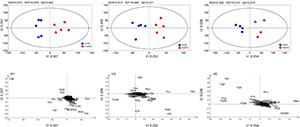
Score plots and loading diagram of orthogonal partial least squares discrimination analysis (OPLS-DA) metabolic profiles in the needles of *A. faxoniana* at 3,500, 3,000, and 2,600 m under controls and OTCs. H1, the altitude of 3,500 m; H2, the altitude of 3,000 m; H3, the altitude of 2,600 m.

In this study, 71 metabolites were identified by GC–MS in the needles of *A. faxoniana* (Supplementary Table 1), mainly including amino acids, organic acid, and sugars. However, a large number of metabolites cannot be positively identified by using GC–MS, especially secondary metabolites. To visually present the altitude and warming effects, the control and warming groups of the same altitude were divided into the same clusters in the PLS-DA, indicating a clear altitude effect (Supplementary Figure 4). The results of the OPLS-DA indicated that the control and warming samples at each altitude could be clearly separated (Figure 4). Organic acids, sugars, and amino acids were greatly influenced by warming (Figure 4). A heatmap of the 28 samples also showed that the most important factor to causing the obvious separation was altitude but not warming (Supplementary Figure 3).

### Different Metabolites Between Altitudes Under Open-Top Chamber Warming Conditions

There were 28, 15, and 17 metabolites significantly affected by altitude, warming, and their interaction (Supplementary Table 1), while 23, 12, and 15 metabolites could be considered as DEMs, respectively (Table 3). Under ambient conditions, there were less increased than decreased metabolites compared between low and high altitude. Of these, the contents of most of amino acids and sugars (except sucrose) were higher at high than low altitudes. OTC warming significantly increased the abundances of most of the metabolites at 2,600 m but the trend is the opposite at 3,500 m, e.g., organic acids, ribose, uracil, urea, and threitol.

**TABLE 3:** The significant fold changes of differentially expressed metabolites (DEMs) in needles of *A. faxoniana* as affected by altitude and warming.

	**H3C/H2C (2,600/3,000)**	**H2C/H1C (3,000/3,500)**	**H3C/H1C (2,600/3,500)**	**H3O/H3C (2,600)**	**H2O/H2C (3,000)**	**H1O/H1C (3,500)**	***P*_*a*_**	***P*_*t*_**	***P*_*a* ×_ *_*t*_***
**Sugars**									
Fructose-6-phosphate	0.97	**0.66**	**0.64**	0.89	**0.72**	**0.70**	0.00	0.00	0.31
Fructose	0.96	**0.78**	**0.75**	1.01	1.09	1.03	0.00	0.20	0.61
Glucose	1.03	**0.75**	**0.77**	0.92	**1.23**	1.00	0.00	0.28	0.02
Glucose-6-phosphate	0.91	**0.66**	**0.60**	0.93	**0.56**	**0.70**	0.00	0.00	0.20
Arabinose	1.06	0.93	1.00	1.05	1.03	**0.87**	0.07	0.49	0.04
Sucrose	**1.16**	1.11	**1.30**	0.90	1.08	**1.21**	0.13	0.27	0.04
Xylose	0.84	**0.57**	**0.48**	0.88	1.36	**0.64**	0.10	0.60	0.21
**Amino acids**									
Beta-alanine	0.62	0.82	**0.51**	1.53	0.94	**0.59**	0.33	0.50	0.02
Serine	0.67	0.74	**0.49**	1.48	1.19	0.99	0.00	0.13	0.43
Phenylalanine	**0.46**	**0.65**	**0.30**	2.21	1.49	**0.73**	0.01	0.16	0.02
Leucine	0.61	1.35	**0.83**	2.43	0.53	0.84	0.68	0.78	0.28
Iso-leucine	**0.37**	1.39	0.52	1.78	1.04	0.74	0.09	0.80	0.61
Threonine	0.75	0.93	**0.70**	1.52	1.05	0.92	0.78	0.28	0.21
Tyrosine	1.11	**0.61**	**0.67**	0.83	1.49	1.02	0.00	0.31	0.05
Valine	1.43	0.38	0.54	1.34	1.18	**0.25**	0.39	0.27	0.03
Glutamic acid	0.86	1.02	0.88	1.23	0.97	**0.70**	0.24	0.61	0.07
**Secondary metabolites**									
Catechin	1.56	0.83	1.30	**0.22**	**0.56**	1.16	0.02	0.00	0.00
Dehydroascorbic acid dimer	**2.70**	**0.28**	0.76	**0.18**	0.47	0.67	0.00	0.01	0.36
2,3-Dihydroxybutanedioic acid	**0.53**	0.88	**0.46**	1.20	0.73	1.08	0.02	0.86	0.52
*p*-Coumaric acid	1.08	**0.78**	0.84	0.94	1.24	0.98	0.02	0.44	0.10
5-Trans-Caffeoylquinic acid	0.41	0.59	**0.24**	1.48	3.59	2.57	0.48	0.28	0.80
**Organic acids**									
2,4,5-Trihydroxypentanoic acid	0.89	1.03	0.92	**1.49**	1.02	1.34	0.43	0.01	0.14
Gluconic acid	1.52	0.46	0.69	**0.40**	0.94	0.97	0.00	0.17	0.31
Heptanoic acid	**1.79**	0.85	**1.54**	**0.28**	1.20	0.86	0.88	0.00	0.00
Glyceric acid	**0.62**	**0.63**	**0.39**	**1.95**	1.16	**0.81**	0.00	0.10	0.00
Oxalic acid	**0.06**	0.94	**0.06**	**39.12**	0.85	1.03	0.70	0.03	0.01
Quinic acid	0.99	**0.76**	**0.76**	0.86	0.98	**0.76**	0.00	0.01	0.07
Threonic acid	0.87	0.88	0.77	0.56	0.73	1.08	0.03	0.20	0.37
Threonic acid-1,4-lactone	**0.61**	0.71	**0.43**	1.03	1.11	0.82	0.00	0.72	0.37
Lactic acid	0.92	0.46	**0.42**	0.93	1.27	0.95	0.01	0.92	0.86
Malonic acid	0.81	1.25	1.02	**2.09**	0.39	0.98	0.10	0.67	0.03
Succinic acid	0.95	0.93	0.90	0.63	0.92	**0.86**	0.28	0.08	0.60
**Others**									
1-Methyl-beta-D-mannopyranoside	1.56	**0.60**	0.94	0.75	1.67	1.06	0.15	0.49	0.07
Arabitol	1.14	2.94	**3.33**	0.99	1.96	0.92	0.17	0.55	0.65
Ethanolamine	1.18	**0.65**	**0.77**	0.82	1.06	**0.82**	0.00	0.05	0.10
Hydroxylamine	**10.00**	0.29	2.94	1.02	1.18	1.55	0.00	0.68	0.91
Monomethylphosphate	1.00	**1.92**	1.92	1.28	0.57	1.37	0.04	0.92	0.13
Myo-inositol	0.90	**0.65**	**0.58**	0.57	0.98	0.90	0.00	0.02	0.16
Ribose	0.79	0.86	**0.68**	**1.73**	1.10	0.97	0.67	0.02	0.03
Threitol	0.83	**0.65**	**0.54**	**4.73**	1.39	**0.73**	0.00	0.00	0.00
Uracil	**0.75**	0.88	**0.66**	**1.70**	0.99	1.01	0.21	0.03	0.02
Urea	**0.71**	**0.53**	**0.38**	**1.57**	1.08	0.68	0.00	0.80	0.03

*P_a_, altitude effect; *P*_*o*_, OTC effect; *P*_*a* ×_ *_*o*_*, altitude and OTC interaction effect; H1C, 3,500 m control; H2C, 3,000 m control; H3C, 2,600 m control; H1O, 3,500 m OTC warming; H2O, 3,000 m OTC warming; H3O, 2,600 m OTC warming; bold number, there was significantly difference between two treatments according to *t*-test (*p* < 0.05).*

**FIGURE 5:**
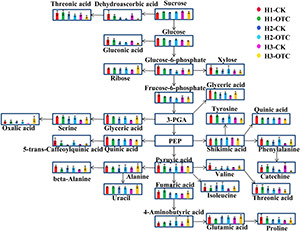
Metabolic pathways in needles of *A. faxoniana* at 3,500, 3,000, and 2,600 m caused by warming. Values were used for intensities of the detected metabolites. H1, the altitude of 3,500 m; H2, the altitude of 3,000 m; H3, the altitude of 2,600 m. PEP, phosphoenolpyruvic acid; 3-PGA, 3-phosphoglyceric acid.

For sugars, the concentrations of fructose-6-phosphate (F6P), glucose-6-phosphate (G6P), and xylose were significantly decreased by warming at 3,500 m but were not decreased at 2,600 m (Figure 5) while sucrose and ribose significantly increased, and xylose decreased at three altitudes. The most significant changes were found in amino acids. Beta-alanine, serine, phenylalanine, leucine, iso-leucine, threonine, valine, and glutamic acid were increased at 2,600 m but most of them were decreased at 3,500 m. Additionally, organic acids also showed different altitude-related variation. Among these metabolites, the most significant increase was found in oxalic acids, with an increase of almost 39-fold at 2,600 m, while the most significant decrease was found in valine, with a decrease of one fourth at 3,500 m by warming (Figure 5).

### Correlation Between the Ionome and Metabolome

**FIGURE 6:**
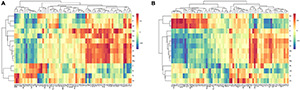
The heat maps of the correlation between soil mineral elements and needle metabolites in CK groups **(A)** and OTC groups **(B)**. Horizontal axis indicates the needle metabolites with significant changes under warming conditions; vertical axis indicates the needle mineral elements with significant changes warming conditions. Abbreviations were listed in Supplementary Table 1.

**FIGURE 7:**
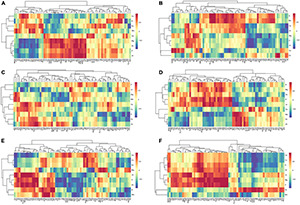
The heat maps of the correlation between needle mineral elements and needle metabolites in CK groups and OTC groups at the three altitudes. **(A)**, H1CK; **(B)**, H1OTC; **(C)** H2CK; **(D)** H2OTC; **(E)** H3CK; and **(F)** H3OTC. Horizontal axis indicates the needle metabolites with significant changes under warming conditions; vertical axis indicates the needle mineral elements with significant changes warming conditions. CK, control groups; OTC, warming groups. H1, 3,500 m; H2, 3,000 m; H3, 2,600 m. Abbreviations were listed in Supplementary Table 1.

Comprehensive analysis with the Pearson correlation coefficient were conducted to understand the relationships between soil mineral elements and needle metabolites (Figure 6). Under warming conditions, Na, K, Mg, Al, Zn, Fe, and P were positively correlated with phenylalanine and bata-alanine. The correlations among oxalic acid and P, Fe, Na, and K were negative. Fumaric acid and malic acid were positively correlated with Zn, Fe, K, Na, and Al at ambient conditions, while the correlations were negative after OTC warming. Moreover, the correlations of needle nutrients and metabolites at different altitudes were also explored. Under warming conditions, P was positively correlated with amino acids (threonine, serine, and valine), benzoic acid, G6P, and citric acid at 2,600 m (Figure 7). In addition, oxalic acid, sucrose, and phenylalanine were positively correlated with Al, and the correlation between quinic acid and Mn was increased at 2,600 m under OTC warming (Figure 7). However, P was negatively correlated to the tyrosine, leucine, proine, shikimic acid, oxalic acid, fructose, G6P, and fumaric acid while Al was negatively correlated with malic acid, leucine, glucose, and oxalic acid at 3,500 m. In addition, Mn was negatively correlated with many metabolites involved in carbohydrate metabolism such as malic acid, oxalic acid, fructose, citric acid, fumaric acid, glucose, glycerol, and shikimic acid at 3,500 m (Figure 7).

## Discussion

### The Effects of Altitude and Long-Term Warming on Soil Nutrients

Different altitudes at small geographic scales, such as subalpine ranges, exhibit steep climatic gradients with decreasing temperature (Seastedt and Oldfather, 2021). Forest soil properties differ greatly across altitudes. The soil C:N ratio ranged from 13.49 to 16.77 at three altitudes (Table 2), which was higher than the average value (12.30) in China (Tian et al., 2010). When soil C:N is less than 25:1, soil nitrogen is sufficient, and organic carbon mineralization occurs more easily (Tessier, 2001). Therefore, regardless of growth conditions, the soil C and N supply could be sufficient at these study sites, and microbes utilize organic matter effectively (Xu et al., 2015). Soil C:P reflects the effectiveness of phosphorus utilization, and N:P is often used for the diagnosis of nutrient limitation of soil N or P. Soil and plant N:P ratios are closely related to each other (Fan et al., 2015). In China, the average C:P and N:P of soil are 52.70 and 3.90, respectively (Tian et al., 2010). Additionally, the deficiencies of P, Ca, and Mg are common with warming in the subalpine regions of western China (Ji et al., 2019; Yuan et al., 2019). In this study, C:P and N:P ratios were much higher in the three altitudes than the average values across China, indicating that soil P availability is relatively low, which is not conducive to plant growth. Warming significantly decreased the soil P concentration and increased the needle P concentration at low altitudes (Table 2), suggesting that soil P would be a main limiting factor for *A. faxoniana* at low altitudes with the increasing global temperature.

The concentrations of soil cations (Ca, Na, Al, Mg, and K) were increased along the altitude. However, OTC warming caused the opposite changes in the soil Na, Al, and Zn between lower and higher altitudes (Table 2). These mineral nutrients come mainly from rock weathering (Uhlig and von Blanckenburg, 2019). Warming can cause more significant changes in the soil microbial community and enzyme activities at low than at high altitude (Zhao et al., 2014; Xu et al., 2015). The microbial activities enhanced by warming at low altitude could accelerate soil nutrient cycling. Compared with the low altitude, the relatively lower annual temperature at high altitude caused a relatively lower rate of nutrient mineralization. At low altitude (2,600 m), the OTC warming effect (1.04°C) was distinctly higher than that at high altitude and would accelerate rock weathering. Therefore, more mineral nutrients, including heavy metals, would be released into the soil under OTC warming at low than high altitude.

### Long-Term Warming Effects in *Abies faxoniana* Saplings at Different Altitudes

Needle δ^13^C can be a good proxy for evaluating long-term water use efficiency of trees (Gimeno et al., 2021). There was no significant change in needle δ^13^C values between the control, and OTCs showed there was no influence on water use efficiency in *A. faxoniana* under long-term warming (Terwilliger et al., 2001), further indicating that water supply was sufficient in the control and warming groups (Figure 1A).

Sugars play important roles in regulating osmotic potential in plants under adverse conditions. Plants have developed an ability to resist cold damage by synthesizing numerous protective substances (e.g., soluble sugars and prolines) and proteins when they are exposed to low temperature (Ding et al., 2019). The metabolic data indicated that the higher content of sugars detected at the high altitude was a sign of greater protection against chilling stress in *A. faxoniana* cells. Sugars also play important roles in energy acquisition, as they are sources of carbon and nitrogen (Larher et al., 2003). Warming can also induce declines in the concentration of starch and transcripts of genes involved in glycolysis, TCA cycle, and secondary metabolism, accompanied by an increase in glucose (Vicente et al., 2019). In our study, F6P, G6P, and xylose were significantly decreased by OTC warming at high altitude but were not decreased at low altitude. F6P and G6P are important compounds in the first steps of glycolysis. G6P is the starting point for energy metabolism and is also used to generate NADPH in plants (Schluepmann et al., 2012). We think that on the one hand, the decreases in F6P and G6P contents can be attributed to the lower efficiency of hexose phosphorylation by hexokinase rather than the reduction in glucose supply. On the other hand, under warming conditions, *A. faxoniana* growing at high altitudes possibly consumes less energy than that growing at low altitude (Zhao et al., 2020; Zhang et al., 2021). Therefore, *A. faxoniana* at low altitude need to obtain more energy to cope with warming, whereas it is most likely to adopt an energy conservation strategy at high altitude.

Among the identified metabolites, the greatest changes caused by OTC warming among altitudes were amino acids. The content of γ-aminobutyric acid (GABA) was significantly increased due to OTC warming. GABA is an essential intermediate in the biosynthesis of many amino acids in plants (Bouché and Fromm, 2004; Batushansky et al., 2014). The increase of GABA, glutamic acid, and alanine in plants is an essential adaption to temperature elevation (Al-Quraan et al., 2012; Li et al., 2020). Many amino acids were significantly increased at 2,600 m (Figure 7). In particular, valine, beta-alanine, glutamic acid, and phenylalanine showed opposite trends due to OTC warming between the low and high altitudes. Moreover, long-term warming also significantly changed the levels of organic acids in the needles. At low altitude, glyceric acid, malonic acid, and oxalic acid were significantly increased in needles, whereas gluconic acid and heptanoic acid were significantly decreased (Figure 7). Notably, the level of oxalic acid was increased greatly at low but not at high altitude. Leaf respiration is commonly positive correlation with the increased temperature (Reich et al., 2016). Therefore, the increase of organic acids related to carbohydrate metabolism possibly indicates the enhanced leaf respiration under warming conditions.

### The Interaction Between Nutrients and Physiological Responses

**FIGURE 8:**
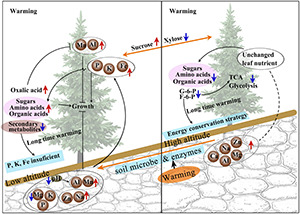
Diagram of *A. faxoniana* responding to warming at low and high altitudes. F6P, fructose-6-phosphate; G6P, glucose-6-phosphate.

Because soils at high altitudes are exposed to harsher environmental conditions, soil mineral nutrition will be different with that in low-altitude areas. Rock weathering and soil microbial community and enzyme activities can be enhanced greatly under warming at low altitudes (Zhao et al., 2014; Xu et al., 2015; Figure 8). In addition, the availability of soil nutrient and elemental composition can greatly affect plant growth and metabolic processes (Rivas-Ubach et al., 2012). Elevation-driven changes in plant nutrients are associated with the content and quality of soil organic matter and the microbial activity (Mayor et al., 2017). For plant growth, Mn is involved in many physiological processes, but only in small quantity (Foy et al., 1978; Millaleo et al., 2010). In contrast, Al is not necessary but will be toxic to plants at high concentration (Delhaize and Ryan, 1995; Kochian et al., 2005). Tree growth was significantly and negatively related to the concentrations of Mn and Al in soil that would have a toxicity effect due to their solubilization (Zemunik et al., 2018). The soil pH can drastically influence the availability of metals (Lambers et al., 2008), and plant accumulation of Mn and Al was likely due to soil acidification at low altitude (Arduini et al., 1998; Yuan et al., 2019; Figure 8). Therefore, only a small increase in height of *A. faxoniana* by warming can be due to a decrease in soil P and an increase in Al and Mn at low altitude.

According to previous studies, plant amino acid metabolism can be improved by temperature change. In addition to osmotic regulation, the increased amino acids also play important roles in protecting plants against pathogenic bacteria and heavy metals (Xie et al., 2013; Sonawala et al., 2018). However, we did not find visible disease symptoms on *A. faxoniana* at 2,600 m, and there was a large decrease in the secondary metabolites including catechin and dehydroascorbic acid. Therefore, the disease effects can be excluded. Warming triggered amino acid alteration can significantly up-regulate stress-related proteins (e.g., chalcone isomerase, glutathione *S*-transferase, and isoflavone reductase) to combat heavy metals (Bilal et al., 2019). Therefore, the increased amino acids by OTC warming at low altitude probably were used to inactivate heavy metals by forming amino acid-cation-polyphosphate complexes to reduce Al and Mn mobility (Figure 8). In addition, oxalic acid can balance the charges of positive ions and amino acids present in the plant cells (Liang et al., 2009; Ma et al., 2011). It has been reported that oxalic acid can form complexes with Al based on the concentration of oxalic acid (Nordstrom and May, 1996; Nakata, 2003). Both the increased internal tolerance and the exclusion of Al from plants are related to oxalate production (Zheng et al., 1998). The increased amount of oxalic acid in needles at low altitudes sequesters Al as non-toxic Al-oxalate salts (Figure 8). Under warming at 2,600 m, amino acid and carbohydrate metabolism related metabolites showed a positive correlation with P, K, Al, and Mn. Importantly, *A. faxoniana* at low altitude have to increase metabolite contents to cope with Al and Mn toxicity. However, concentrations of nutrients in needles of *A. faxoniana* at high altitude did not change along with soil nutrients. Many amino acids, organic acids, F6P, and G6P were decreased at high altitude with OTC warming (Figure 8). Succinic acid, glyceric acid, F6P, and G6P are closely related to glycolysis and TCA cycle. Many amino acids and metabolites involved in carbohydrate metabolism showed negative correlation to P, Al, and Mn concentrations in needles at high altitude. This indicates trees could have less energy production and accumulate more sucrose at high altitude (Figure 8).

Overall, OTC warming can directly affect plant physiological processes and indirectly change soil nutrient conditions, especially of P and heavy metals, and sequentially impact plant growth. Contrary to soil nutrient alterations, cations in *A. faxoniana* needles increased with decreasing altitude. The inconsistent nutrient changes indicated that Ca, Na, Mg, and K in soils were not the limiting nutrients for the growth of *A. faxoniana*. The warming-facilitated exudation of organic acids from plants to soils can reduce the soil pH value further. In addition, trees experience physiological reprogram with increasing age, including decreased growth rates and shifted carbon distribution pattern (Rossi et al., 2008). Younger trees tend to be more sensitive to climate warming, but older trees show remarkable resistance to warming (Colangelo et al., 2021). In this study, 10-year-old young *A. faxoniana* seedlings were used. Therefore, the results might be different from the adult trees at certain extent. In addition, there are some limitations in the current warming method. Compared with infrared heaters, it is unavoidably affected the changes of light, air exchange, and soil microbes by the chambers when using OTC warming. In a word, in the future, although the increased temperature will benefit the high-altitude boundary tree population, it is a disadvantage for the low-altitude boundary population. Therefore, the distribution area of *A. faxoniana* population is unlikely to be enlarged with global warming.

## Conclusion

There are significant altitude effects on the soil properties and tree growth. Warming changes the soil cation concentrations differentially at various altitudes, and the concentrations of metals (e.g., Al and Mn) were increased greatly at low altitudes. Needles of *A. faxoniana* will compete for the mineral nutrients (e.g., Fe, K, and P) at low altitudes in case of warming. The metabolites that differed mostly among the altitudes were the sugars and amino acids. Oxalic acid could be considered an Al and Mn detoxification agent at low altitude. Combined with the growth conditions, we think there are contrasting physiological and metabolic responses of *A. faxoniana* to long-term warming between low and high altitudes, whereby *A. faxoniana* growing at low low-altitude boundary will be more susceptible to climate change in alpine regions.

## Data Availability Statement

The original contributions presented in the study are included in the article/Supplementary Material, further inquiries can be directed to the corresponding author.

## Author Contributions

HS performed the experiments and wrote and revised the manuscript. QH did some lab work. SZ designed the research, analyzed the data, and revised the manuscript. All authors read and approved the final manuscript.

## Conflict of Interest

The authors declare that the research was conducted in the absence of any commercial or financial relationships that could be construed as a potential conflict of interest.

## Publisher’s Note

All claims expressed in this article are solely those of the authors and do not necessarily represent those of their affiliated organizations, or those of the publisher, the editors and the reviewers. Any product that may be evaluated in this article, or claim that may be made by its manufacturer, is not guaranteed or endorsed by the publisher.

## Footnotes

1
http://www.nist.gov/index.html

2
http://www.genome.jp/kegg/

3
www.bioconductor.org/

4
http://www.umetrics.com/simca

5
http://www.metaboanalyst.ca/faces/ModuleView.xhtml

## Supplementary Material

Supplementary Table 1 The identified metabolites in needles of *A. faxoniana* at 3500, 3000 and 2600 m caused by warming. H1, the altitude of 3500 m; H2 the altitude of 3000 m; H3, the altitude of 2600 m.
