# Supplementary material for: Low-Altitude Boundary of Abies faxoniana Is More Susceptible to Long-Term Open-Top Chamber Warming in the Eastern Tibetan Plateau
Source: Front Plant Sci. 2021 Dec 3;12:766368. doi: 10.3389/fpls.2021.766368 (PMC8678095; doi:10.3389/fpls.2021.766368)

Supplemental Fig. S1. The study sites (A) and diagrammatic sketch of planted model (B) of *A. faxoniana* by OTC warming in Wanglang National Nature Reserve. The middle planted sampling (red star) was used for sample collection for metabolome and ionome analyses.

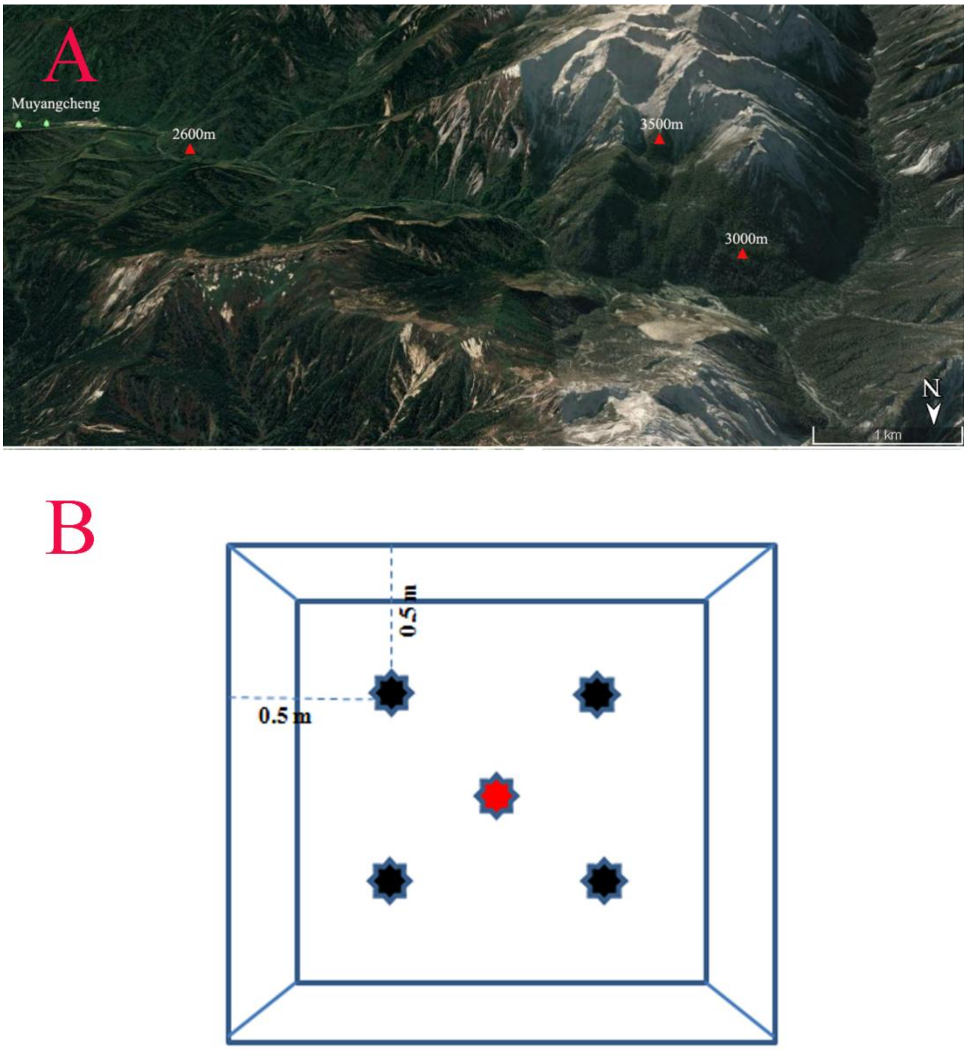

Supplemental Fig. S2. Mean air temperature variation in control and OTCs at altitudes of 3500 (a), 3000 (b) and 2600 m (c) in January to December. X-axis, the mean daily air temperature at the height of 20 cm; Y-axis, temperature.

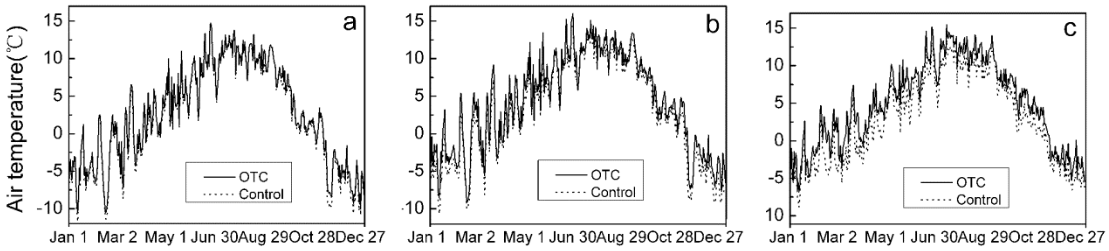

Supplemental Fig. S3. Heatmap analysis for the mineral nutrition (A) and metabolites (B) in needles of *A. faxoniana* at 3500, 3000 and 2600 m under controls and OTCs. H1, the altitude of 3500 m; H2 the altitude of 3000 m; H3, the altitude of 2600 m. Abbreviations were listed in Supplemental Table S1.

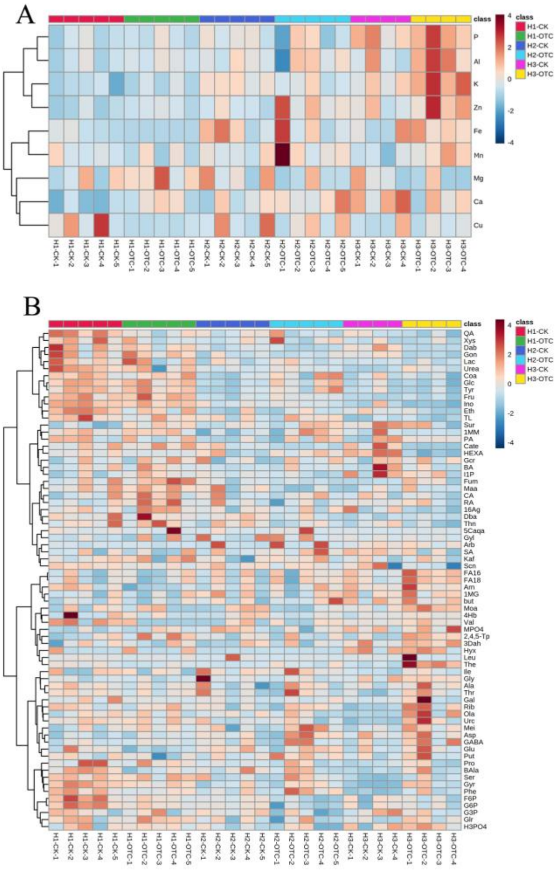

Supplemental Fig. S4. Score plots of PLS-DA of metabolic profiles in the needles of *A. faxoniana* at 3500, 3000 and 2600 m under controls and OTCs. H1, the altitude of 3500 m; H2 the altitude of 3000 m; H3, the altitude of 2600 m.

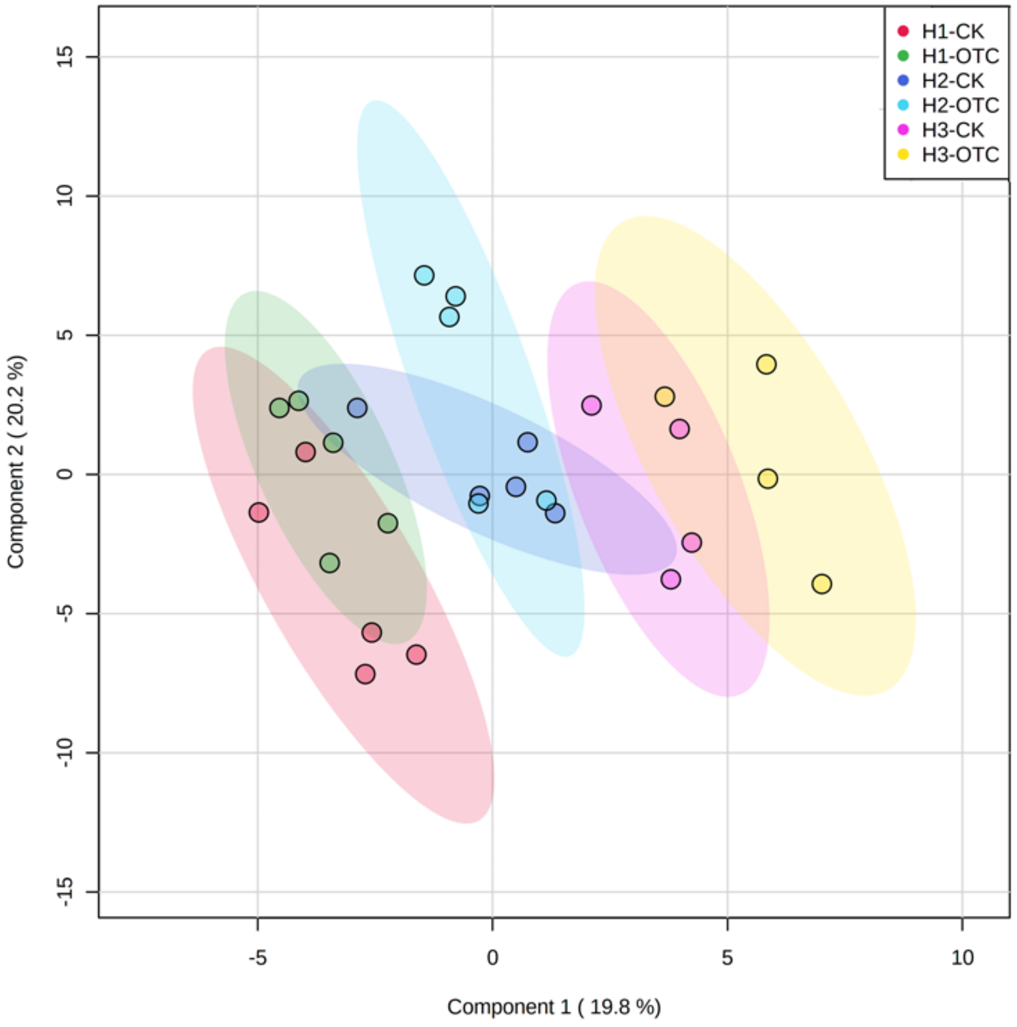

Supplement: Supplementary file 2 [file Data_Sheet_1.PDF]
